# Synthesis of *gem*-difluoromethylenated analogues of boronolide

**DOI:** 10.3762/bjoc.6.37

**Published:** 2010-04-20

**Authors:** Jing Lin, Xiao-Long Qiu, Feng-Ling Qing

**Affiliations:** 1 College of Chemistry, Chemical Engineering and Biotechnology, Donghua University, 2999 North Renmin Lu, Shanghai 201620, China; 2 Key Laboratory of Organofluorine Chemistry, Shanghai Institute of Organic Chemistry, Chinese Academy of Sciences, 345 Lingling Lu, Shanghai 200032, China

**Keywords:** boronolide, *gem*-difluoromethylenated analogues, *gem*-difluoropropargylation, α,β-unsaturated-δ-lactones

## Abstract

The straightforward synthesis of four *gem*-difluoromethylenated analogues **4**–**7** of boronolide is described. The key steps of the synthesis include the concise preparation of the key intermediates **12a**–**b** through the indium-mediated *gem*-difluoropropargylation of aldehyde **9** with the fluorine-containing building block **11** and the efficient construction of α,β-unsaturated-δ-lactones **15a**–**b** via BAIB/TEMPO-procedure.

## Introduction

**Figure 1:**
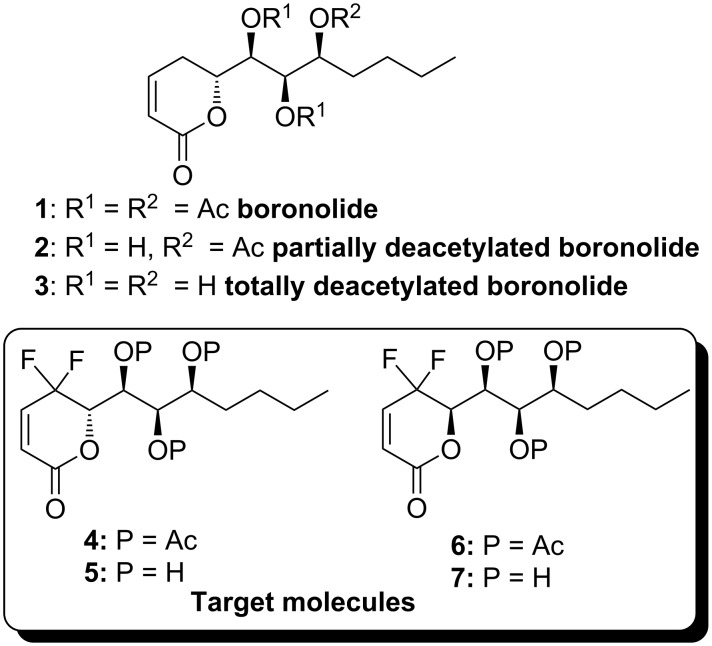
Boronolide (**1**), boronolide analogues **2**–**3** and *gem*-difluoromethylenated analogues **4**–**7**.

(+)-Boronolide (**1**), isolated from the bark and branches of *Tetradenia fruticosa* [1] and from the leaves of *Tetradenia barberae* [2], has been used as a traditional medicine in Madagascar and South Africa [2–4]. In addition, a partially deacetylated analogue **2** and the totally deacetylated analogue **3** have also been obtained from *Tetradenia riparia* [3,5], a Central African species traditionally employed by the Zulu as an emetic, and whose leaf infusions have also been reported to be effective against malaria [2,4]. Boronolide (**1**) and its analogues **2**–**3** feature an interesting polyacetoxylated (or polyhydroxyl) side chain and an α,β-unsaturated-δ-lactone moiety, making them an attractive target for total syntheses [6–16] since many natural products with a wide range of biological activity contain these structural elements. Noteworthily, structure–activity relationships have demonstrated that the α,β-unsaturated-δ-lactone moiety plays a key role in the bioactivity of many natural products. This is due to the fact that this unit is an excellent potential Michael acceptor for nucleophilic amino acid residues of the natural receptors interacting with these compounds [16–18]. Considering the similarity in size of fluorine and hydrogen atoms, the strong electron-withdrawing property of *gem*-difluoromethylene group (CF_2_) [19–20] and our continual efforts to prepare *gem*-difluoromethylenated analogues of natural products containing α,β-unsaturated-δ-lactone moiety [21–24], we intended to introduce a CF_2_ group to α,β-unsaturated-δ-lactone of boronolide at the γ-position (Figure 1). We envisioned that the resulting γ,γ-difluoromethylenylated–α,β-unsaturated conjugated double bond would be much more electron-deficient and therefore a better candidate to enhance the reactivity of the conjugated double bond as an acceptor with minimum steric change. In this article we describe the concise synthesis of *gem*-difluoromethylenated analogues **4**–**7** of boronolide.

## Results and Discussion

**Scheme 1:**
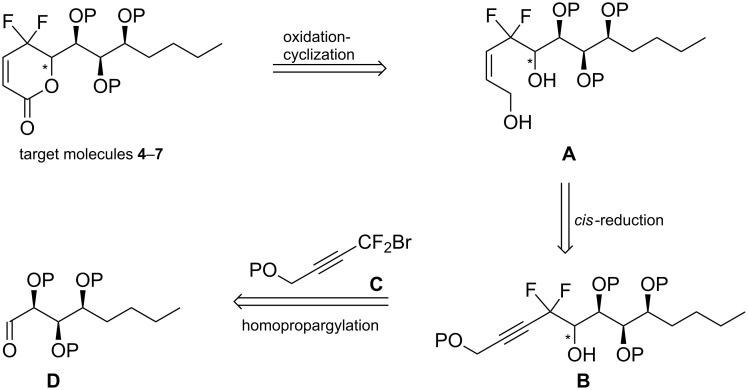
Retrosynthetic analysis of target molecules **4**–**7**.

The retrosynthetic analysis of the target molecules **4**–**7** is outlined in Scheme 1. We envisioned that the key γ,γ-*gem*-difluoromethylenated α,β-unsaturated-δ-lactone scaffold could be constructed via an oxidation–cyclization reaction of intermediate **A** according to our published procedures [22]. *cis*-Selective reduction of homopropargyl alcohol **B** would provide the homoallylic alcohol **A**. The alcohol **B** could be obtained via an indium-mediated reaction of the fluorine-containing building block **C** and the protected aldehyde **D**, which in turn could be readily prepared by the reported procedure. Three chiral centres in our target molecules would be derived from D-glucono-δ-lactone, and the last one could be constructed by diastereoselective propargylation of the aldehyde.

**Scheme 2:**
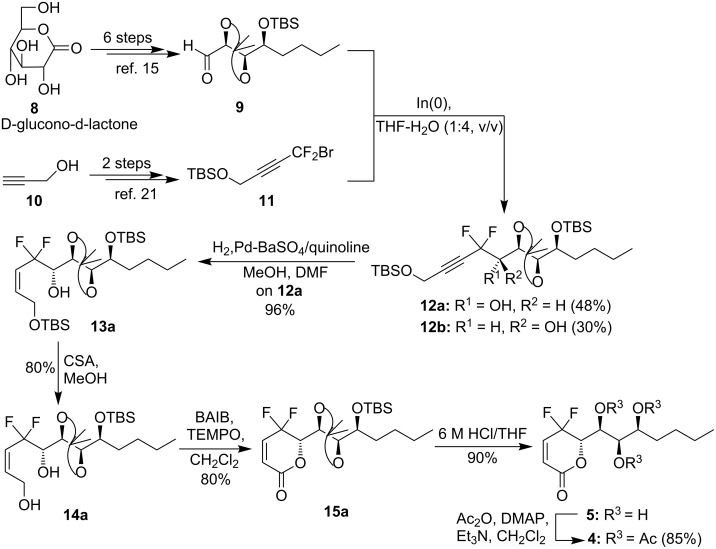
Synthesis of target molecules **4**–**5**.

According to the retrosynthetic analysis our synthesis embarked from aldehyde **9**, which was prepared from commercially available D-glucono-δ-lactone (**8**) in six steps, based on the reported route [15] (Scheme 2). The synthesis of the fluorine-containing intermediate **11** was accomplished from propargyl alcohol (**10**) by our improved procedure [22]. With these two key fragments in hand, we focused our efforts on the *gem*-difluoropropargylation reaction. Utilizing Hammond’s reaction conditions [25], we were pleased to find that treatment of aldehyde **9** with compound **11** in the presence of indium with THF–H_2_O (1:4, v/v) as solvent smoothly gave the expected product **12a** in 48% yield and **12b** in 30% yield. More pleasing was the fact that the diastereomers **12a** and **12b** could be easily separated by silica gel chromatography. The assignment of the stereochemistry of the formed alcohol groups in **12a** and **12b** was based on the comparison of the ^19^F NMR spectra of compounds **5** and **6** with those of our synthesized *gem*-difluoromethylenated goniodiols. The absolute configuration of the formed alcohol was determined by X-ray crystallographic analysis [23]. Initial attempts to convert the triple bond in compound **12a** into the *cis* double bond via hydrogenation in the presence of Lindlar catalyst were unsuccessful. Even with the addition of quinoline, these reactions only resulted in inseparable mixtures. Fortunately, hydrogenation proceeded well by means of Pd–BaSO_4_–quinoline system [26], leading to the expected alcohol **13a** in 96% yield. Subsequent selective removal of the primary TBS group in **13a** with D-camphor-10-sulfonic acid (CSA) yielded the diol **14a** in 80% yield. As expected, treatment of compound **14a** with 0.2 equiv of 2,2,6,6-tetramethyl-1-piperidinyloxy (TEMPO) and 3.0 equiv of [bis(acetoxy)iodo]benzene (BAIB) in dichloromethane at room temperature afforded the desired α,β-unsaturated-δ-lactone **15a** in 80% yield. Exposure of compound **15a** to a solution of 6 M HCl in aqueous THF removed the protecting groups smoothly, and the deacetylated boronolide derivative **5** was obtained in 90% yield. Additionally, *gem*-difluoromethylenated boronolide **4** was prepared in good yield via treatment of compound **5** with Ac_2_O/DMAP/Et_3_N.

**Scheme 3:**
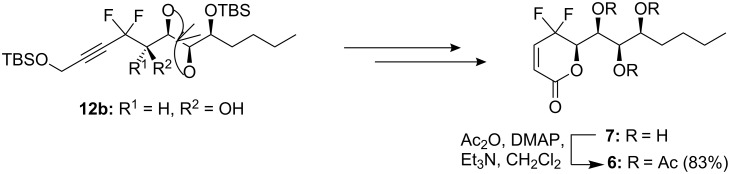
Synthesis of target molecules **6**–**7**.

Using similar reaction conditions, *gem*-difluoromethylenated boronolide analogues**6**–**7** were also synthesized from the intermediate **12b** (Scheme 3).

## Conclusion

In summary, we accomplished a concise synthesis of the *gem*-difluoromethylenated analogues of boronolide **4**–**7**. Our approach featured the preparation of separable key diastereoisomers **12a**–**b** from the indium-mediated *gem*-difluoropropargylation of aldehyde **9** with fluorine-containing building block **11** and the efficient construction of α,β-unsaturated-δ-lactones **15a**–**b** via the BAIB/TEMPO-procedure.

## Supporting Information

Supporting information features synthesis and characterization of *gem*-difluoromethylenated analogues of boronolide:

File 1 Synthesis, characterization of concerned compounds.
