# Supplementary material for: Synthesis of gem-difluoromethylenated analogues of boronolide
Source: Beilstein J Org Chem. 2010 Apr 20;6:37. doi: 10.3762/bjoc.6.37 (PMC2874314; doi:10.3762/bjoc.6.37)
Supplement: File 1 — Synthesis, characterization of concerned compounds. [file Beilstein_J_Org_Chem-06-37-s001.pdf]

Supporting Information

for

Synthesis of *gem*-difluoromethylenated analogues of boronolide

Jing Lin[1], Xiao-Long Qiu[2] and Feng-Ling Qing*[1,2]

Address: [1]College of Chemistry, Chemical Engineering and Biotechnology, Donghua University, 2999 North Renmin Lu, Shanghai 201620, China and [2]Key Laboratory of Organofluorine Chemistry, Shanghai Institute of Organic Chemistry, Chinese Academy of Sciences, 345 Lingling Lu, Shanghai 200032, China

Email: Feng-Ling Qing - flq@mail.sioc.ac.cn

* Corresponding author

Experimental section

Unless otherwise indicated, all chemicals and solvents were used as received from commercial sources or purified by standard procedures. Optical rotations were recorded on a Jasco P-1030 polarimeter. IR spectra were scanned with a Bio-Rad FTS185 spectrophotometer. ^{1}H and ^{13}C NMR spectra were obtained using a Bruker AM300 and a Bruker AM400 spectrometer, respectively. ^{19}F NMR spectra were recorded on a Bruker AM300 spectrometer ($CFCl_3$ as external standard and low field is positive; chemical shifts (δ) are given in ppm and coupling constants (*J*) in Hz). LRMS were measured on an Agilent system mass spectrometer and HRMS on an APEXIII (7.0 Tesla) FTMS or Waters mass spectrometer. Elemental analyses were performed on a Vario EL III elementary analysis instrument.

(*S*)-5-(*tert*-Butyldimethylsilyloxy)-1-((2*S*,3*S*)-3-((*S*)-1-(*tert*-butyldimethylsilyloxy)pentyl)-2,2-dimethyl-1,3-dioxolan-4-yl)-2,2-difluoropent-3-yn-1-ol (12a) and (*R*)-5-(*tert*-butyldimethylsilyloxy)-1-((2*S*,3*S*)-3-((*S*)-1-(*tert*-butyldimethylsilyloxy)pentyl)-2,2-dimethyl-1,3-dioxolan-4-yl)-2,2-difluoropent-3-yn-1-ol (12b)

To a stirred suspension of compound **9** and compound **11** (1.2 g, 4.01 mmol) in THF–H_2O (50 mL, 1:4, v/v), indium powder (400 mg, 3.48 mmol) was added at room temperature. After stirring for 22 h, the reaction mixture was quenched with 1 M HCl. The aqueous phase was extracted with EtOAc, the organic phase washed successively with water and brine, dried over anhydrous Na_2SO_4, filtered and concentrated. The residue was purified by flash chromatography on silica gel (petroleum ether/ethyl acetate = 50:1) to afford **12a** (0.8 g, 48% yield) and **12b** (0.5 g, 30% yield).

12a: yellow oil; $[\alpha]_D^{27} = -3.2$ (*c* 1.00, $CHCl_3$); ^{1}H NMR (300 MHz, $CDCl_3$) δ 4.40 (t, *J* = 4.8 Hz, 2H), 4.18 (t, *J* = 7.8 Hz, 1H), 4.05 (dd, *J* = 7.5 Hz, 3.0 Hz, 1H), 3.92 (m, 1H), 3.84 (m, 1H), 1.75 (m, 1H), 1.50 (m, 1H), 1.47 (s, 3H), 1.43 (s, 3H), 1.34 (m, 4H), 0.91 (m, 21H), 0.13 (s, 12H); ^{13}C NMR (100 MHz, $CDCl_3$) δ 113.4 (t, *J* = 236.6 Hz), 109.7, 87.7, 81.6, 75.5 (t, *J* = 38.5 Hz), 74.9, 74.4 (t, *J* = 28.2 Hz), 71.8, 51.3, 32.6, 28.4, 26.9, 25.9, 25.7, 22.8, 18.2, 18.1, 14.0, −4.5, −4.7, −5.2; ^{19}F NMR (282 MHz, $CDCl_3$) δ −92.1 (dm, *J* = 280.3 Hz, 1F), −94.1 (dm, *J* = 286.2 Hz, 1F); IR (thin film) ν_{max} 3450, 2958, 2260, 1473, 1386, 1256, 837 cm^{-1}; MS (ESI) *m*/*z* 551 $(M+H)^+$; HRMS Calcd for $C_{27}H_{52}F_2O_5Si_2Na$: 573.3214; found: 573.3224.

12b: yellow oil; $[\alpha]_D^{27}$ = −3.3 (*c* 1.00, $CHCl_3$); ^1H NMR (300 MHz, $CDCl_3$) δ 4.40 (t, *J* = 4.8 Hz, 2H), 4.29 (d, *J* = 8.1 Hz, 1H), 4.00 (dd, *J* = 9.0 Hz, 4.2 Hz, 1H), 3.82 (m, 2H), 1.65 (m, 1H), 1.44 (s, 6H), 1.36 (m, 5H), 0.92 (m, 21H), 0.14 (s, 6H), 0.08 (s, 6H); ^{13}C NMR (100 MHz, $CDCl_3$) δ 113.1 (t, *J* = 237.5 Hz), 110.1, 88.1, 79.5, 75.8 (t, *J* = 38.9 Hz), 73.2, 71.7 (t, *J* = 28.2 Hz), 51.2, 32.4, 28.0, 27.3, 26.8, 25.9, 25.7, 22.7, 18.2, 18.1, 14.0, −4.3, −4.7, −5.3; ^{19}F NMR (282 MHz, $CDCl_3$) δ −94.2 (dm, *J* = 276.9 Hz, 1F), −96.3 (dm, *J* = 275.8 Hz, 1F); IR (thin film) ν_{max} 3350, 2933, 2258, 1478, 1386, 1256, 836 cm^{-1}; MS (ESI) *m*/*z* 551 $(M+H)^+$; HRMS Calcd for $C_{27}H_{52}F_2O_5Si_2Na$: 573.3214; found: 573.3237.

(*S*,*Z*)-5-(*tert*-Butyldimethylsilyloxy)-1-((4*R*,5*S*)-5-((*R*)-1-(*tert*-butyldimethylsilyloxy)-pentyl)-2,2-dimethyl-1,3-dioxolan-4-yl)-2,2-difluoropent-3-en-1-ol (13a)

To a solution of Pd-$BaSO_4$ (42 mg, 42 mg/mmol) in MeOH (15 mL), a solution of quinoline (42 mg, 42 mg/mmol) in MeOH (2 mL) was added at 0 °C. After warming to room temperature, the suspension was stirred for 15 min before compound **12a** (550 mg, 1.00 mmol) in DMF (10 mL) was added. After stirring for another 7 h at 35 °C under hydrogen atmosphere (1 atm), ^{19}F NMR indicated the absence of the starting material **12a**. The reaction mixture was filtered and concentrated. The residue was purified by flash chromatography on silica gel (petroleum ether/ethyl acetate = 50:1) to afford compound **13a** (0.53 g, 96% yield) as a yellow oil.

$[\alpha]_D^{27}$ = −4.0 (*c* 1.00, $CHCl_3$); ^1H NMR (300 MHz, $CDCl_3$) δ 5.95 (m, 1H), 5.60 (m, 1H), 4.47 (m, 2H), 4.06 (t, *J* = 7.5 Hz, 1H), 3.98 (dd, *J* = 8.1 Hz, 3.0 Hz, 1H), 3.88 (m, 1H), 3.78 (dt, *J* = 10.5 Hz, 8.7 Hz, 1H), 1.69 (m, 1H), 1.47 (m, 1H), 1.41 (s, 3H), 1.39 (s, 3H) 1.30 (m, 4H), 0.90 (m, 21H), 0.13 (s, 6H), 0.07 (s, 6H); ^{19}F NMR (282 MHz, $CDCl_3$) δ −101.5 (dt, *J* = 259.4 Hz, 11.2 Hz, 1F), −103.9 (dt, *J* = 255.8 Hz, 11.8 Hz, 1F); IR (thin film) ν_{max} 3350, 2958, 2860, 1469, 1383, 1256, 1084, 837, 776 cm^{-1}; MS (ESI) *m*/*z* 553 $(M+H)^+$; Anal. Calcd for $C_{24}H_{46}F_2O_4Si$: C, 58.65; H, 9.84; found: C, 58.73; H, 9.76.

(*R*,*Z*)-5-(*tert*-Butyldimethylsilyloxy)-1-((4*R*,5*S*)-5-((*R*)-1-(*tert*-butyldimethylsilyloxy)-pentyl)-2,2-dimethyl-1,3-dioxolan-4-yl)-2,2-difluoropent-3-en-1-ol (13b)

To a solution of Pd-$BaSO_4$ (34 mg, 42 mg/mmol) in MeOH (15 mL), a solution of quinoline (34 mg, 42 mg/mmol) in MeOH (2 mL) was added at 0 °C. After warming to room temperature, the suspension was stirred for 15 min before compound **12b** (440 mg, 0.80 mmol) in DMF (10 mL) was added. After stirring for another 3.5 h at 35 °C under hydrogen atmosphere, ^{19}F NMR indicated the absence of the starting material **12b**. The reaction mixture

was filtered and concentrated. The residue was purified by flash chromatography on silica gel (petroleum ether/ethyl acetate = 20:1) to afford compound **13b** (0.41 g, 92% yield) as a yellow oil.

$[\alpha]_D^{27}$ = −6.1 (*c* 1.00, $CHCl_3$); ^{1}H NMR (300 MHz, $CDCl_3$) δ 5.98 (m, 1H), 5.51 (m, 1H), 4.47 (m, 2H), 4.21 (d, *J* = 8.7 Hz, 1H), 3.98 (dd, *J* = 8.7 Hz, 4.2 Hz, 1H), 3.75 (m, 2H), 1.63 (m, 1H), 1.42 (s, 6H), 1.35 (m, 5H), 0.90 (m, 21H), 0.08 (s, 12H); ^{19}F NMR (282 MHz, $CDCl_3$) *δ* −101.8 (dt, *J* = 251.3 Hz, 12.4 Hz, 1F), −103.8 (dt, *J* = 259.4 Hz, 12.1 Hz, 1F); IR (thin film) ν_{max} 3350, 2958, 2860, 1472, 1382, 1255, 1082, 837, 775 cm^{-1}; MS (ESI) *m*/*z* 553 $(M+H)^+$; Anal. Calcd for $C_{24}H_{46}F_2O_4Si$: C, 58.65; H, 9.84; found: C, 58.97; H, 9.84.

(*S*,*Z*)-5-((4*R*,5*S*)-5-((*R*)-1-(*tert*-Butyldimethylsilyloxy)pentyl)-2,2-dimethyl-1,3-dioxolan-4-yl)-4,4-difluoropent-2-ene-1,5-diol (14a)

To a solution of **13a** (440 mg, 0.80 mmol) in MeOH (20 mL), CSA (9 mg, 0.02 mmol) was added. After stirring for 1 h, the reaction mixture was quenched with saturated $NaHCO_3$. The aqueous phase was extracted with EtOAc, organic phase washed with brine, dried over anhydrous Na_2SO_4, filtered, concentrated and purified by flash chromatography on silica gel (petroleum ether/ethyl acetate = 4:1) to give **14a** (279 mg, 80% yield) as a clear oil.

$[\alpha]_D^{27}$ = −6.5° (*c* 1.00, $CHCl_3$); ^{1}H NMR (300 MHz, $CDCl_3$) δ 6.08 (m, 1H), 5.68 (m, 1H), 4.37 (m, 2H), 4.04 (t, *J* = 7.5 Hz, 1H), 3.99 (m, 1H), 3.93 (m, 1H), 3.80 (dt, *J* = 10.2 Hz, 7.8 Hz, 1H), 2.29 (brs, 2H), 1.72 (m, 1H), 1.46 (m, 2H), 1.41 (s, 3H), 1.40 (s, 3H) 1.33 (m, 3H), 0.91 (m, 12H), 0.13 (s, 6H); ^{19}F NMR (282 MHz, $CDCl_3$) δ −100.4 (dd, *J* = 258.0 Hz, 16.6 Hz, 1F), −103.5 (ddd, *J* = 255.8 Hz, 16.1 Hz, 8.7 Hz, 1F); IR (thin film) ν_{max} 3403, 2960, 2862, 1469, 1371, 1256, 1075, 837, 776 cm^{-1}; MS (ESI) *m*/*z* 461 $(M+Na)^+$; Anal. Calcd for $C_{24}H_{46}F_2O_4Si$: C, 57.50; H, 9.19; found: C, 57.57; H, 8.93.

(*R*,*Z*)-5-((4*R*,5*S*)-5-((*R*)-1-(*tert*-Butyldimethylsilyloxy)pentyl)-2,2-dimethyl-1,3-dioxolan-4-yl)-4,4-difluoropent-2-ene-1,5-diol (14b)

Compound **14b** was prepared from compound **13b** (415 mg, 0.75 mmol) using the same conditions as described for compound **14a**; yield: 273 mg (83%); clear oil.

$[\alpha]_D^{27}$ = −8.2 (*c* 1.00, $CHCl_3$); ^{1}H NMR (300 MHz, $CDCl_3$) δ 6.07 (m, 1H), 5.60 (m, 1H), 4.40 (m, 2H), 4.24 (d, *J* = 8.4 Hz, 1H), 3.99 (dd, *J* = 8.7 Hz, 3.9 Hz, 1H), 3.80 (m, 2H), 2.16 (brs, 2H), 1.64 (m, 1H), 1.43 (s, 6H), 1.35 (m, 5H), 0.90 (m, 12H), 0.08 (s, 6H); ^{13}C NMR (100 MHz, $CDCl_3$) δ 138.4, 121.8 (t, *J* = 26.0 Hz), 120.2 (t, *J* = 243.8 Hz), 109.5, 78.8, 72.7, 71.1, 70.6 (t, *J* = 29.8 Hz), 58.5, 32.1, 27.6, 26.8, 26.4, 25.4, 22.3, 17.6, 13.5, −4.8, −5.0; ^{19}F NMR

(282 MHz, $CDCl_3$) δ −101.8 (ddd, *J* = 256.6 Hz, 15.5 Hz, 8.7 Hz, 1F), −103.8 (dt, *J* = 258.0 Hz, 15.8 Hz, 1F); IR (thin film) ν_{max} 3350, 2958, 2860, 1469, 1383, 1256, 1084, 837, 776 cm^{-1}; MS (ESI) *m*/*z* 461 $(M+Na)^+$; HRMS Calcd for $C_{21}H_{40}F_2O_5SiNa$: 461.2505; found: 461.2486.

(*S*)-6-((4*S*,5*S*)-5-((*R*)-1-(*tert*-Butyldimethylsilyloxy)pentyl)-2,2-dimethyl-1,3-dioxolan-4-yl)-5,5-difluoro-5,6-dihydropyran-2-one (15a)

To a solution of **14a** (230 mg, 0.53 mmol) in CH_2Cl_2 (15 mL), BAIB (508 mg, 1.57 mmol) and TEMPO (16 mg, 20 mol %) was added at room temperature. After stirring for 3 h, the reaction mixture was quenched with saturated aqueous $Na_2S_2O_3$ and extracted with CH_2Cl_2. The combined organic extracts were washed successively with saturated $NaHCO_3$ and brine, dried over anhydrous Na_2SO_4, filtered and concentrated. The residue was purified by flash chromatography on silica gel (petroleum ether/ethyl acetate = 20:1) to afford **15a** (184 mg, 80% yield) as a white solid, mp = 88–90 °C.

$[\alpha]_D^{27}$ = 60.7 (*c* 1.00, $CHCl_3$); ^{1}H NMR (300 MHz, $CDCl_3$) δ 6.81 (m, 1H), 6.31 (d, *J* = 10.5 Hz, 1H), 4.60 (dt, *J* = 18.6 Hz, 6.3 Hz, 1H), 4.58 (t, *J* = 6.3 Hz, 1H), 4.25 (dd, *J* = 6.0 Hz, 1.5 Hz, 1H), 3.78 (m, 1H), 1.75 (m, 1H), 1.55 (m, 1H), 1.48 (s, 3H), 1.43 (s, 3H), 1.34 (m, 4H), 0.91 (m, 12H), 0.09 (s, 3H), 0.06 (s, 3H); ^{13}C NMR (100 MHz, $CDCl_3$) δ 159.4, 138.1 (dd, *J* = 41.7 Hz, 34.6 Hz), 126.1 (t, *J* = 12.7 Hz), 112.2 (t, *J* = 324.6 Hz), 110.9, 80.4, 79.8 (t, *J* = 37.5 Hz), 72.8, 71.6, 34.1, 27.7, 27.4, 27.1, 25.9, 22.9, 18.2, 14.0, −4.1, −4.8; ^{19}F NMR (282 MHz, $CDCl_3$) δ −106.1 (dd, *J* = 292.2 Hz, 18.1 Hz, 1F), −107.9 (dd, *J* = 293.2 Hz, 6.2 Hz, 1F); IR (thin film) ν_{max} 2933, 2860, 1757, 1653, 1460, 1384, 1255, 834, 777 cm^{-1}; MS (ESI) *m*/*z* 435 $(M+H)^+$, 457 $(M+Na)^+$; HRMS Calcd for $C_{21}H_{34}F_2O_5SiNa$: 457.2192; found: 457.2179.

(*R*)-6-((4*S*,5*S*)-5-((*R*)-1-(*tert*-Butyldimethylsilyloxy)pentyl)-2,2-dimethyl-1,3-dioxolan-4-yl)-5,5-difluoro-5,6-dihydropyran-2-one (15b)

Compound **15b** was prepared from compound **14b** (150 mg, 0.34 mmol) using the same conditions as described for compound **15a**; yield: 126 mg (85%); clear oil.

$[\alpha]_D^{27}$ = 66.8 (*c* 1.00, $CHCl_3$); ^{1}H NMR (300 MHz, $CDCl_3$) δ 6.67 (m, 1H), 6.28 (d, *J* = 9.9 Hz, 1H), 4.81 (d, *J* = 15.0 Hz, 1H), 4.31 (dd, *J* = 9.0 Hz, 2.4 Hz, 1H), 4.21 (dd, *J* = 9.0 Hz, 4.2 Hz, 1H), 3.90 (dt, *J* = 8.1 Hz, 3.9 Hz, 1H), 1.70 (m, 1H), 1.46–1.31 (m, 11H), 0.91 (m, 12H), 0.11 (s, 3H), 0.10 (s, 3H); ^{19}F NMR (282 MHz, $CDCl_3$) δ −85.2 (dd, *J* = 291.3 Hz, 14.4 Hz, 1F), −113.1 (dd, *J* = 290.7 Hz, 8.5 Hz, 1F); IR (thin film) ν_{max} 2934, 2857, 1755,

1460, 1384, 1252, 834, 777 cm^{-1}; MS (ESI) *m/z* 435 $(M+H)^+$, 457 $(M+Na)^+$; Anal. Calcd for $C_{24}H_{46}F_2O_4Si$: C, 58.04; H, 8.35; found: C, 58.51; H, 8.40.

(*S*)-5,5-Difluoro-6-((1*S*,2*R*,3*S*)-1,2,3-trihydroxyheptyl)-5,6-dihydropyran-2-one (5)

To a solution of **15a** (120 mg, 0.28 mmol) in THF (10 mL), HCl (6 M, 5 mL) was added at room temperature. After the absence of the starting material, the reaction was quenched with saturated aqueous $NaHCO_3$. The aqueous phase was extracted with EtOAc, the organic phase washed with brine, dried over anhydrous Na_2SO_4, filtered, concentrated and purified by flash chromatography on silica gel (petroleum ether/ethyl acetate = 1:1) to afford **5** (70 mg, 90% yield) as a white solid, mp = 88–90 °C.

$[\alpha]_D^{28}$ = 44.3° (*c* 0.50, $CHCl_3$); ^{1}H NMR (300 MHz, $CDCl_3$) δ 6.82 (m, 1H), 6.32 (d, *J* = 9.9 Hz, 1H), 4.75 (dt, *J* = 19.8 Hz, 8.4 Hz, 1H), 4.23 (d, *J* = 8.1 Hz, 1H), 3.84 (m, 2H), 2.57 (brs, 3H), 1.60 (m, 2H), 1.41 (m, 4H), 0.93 (t, *J* = 6.6 Hz, 3H); ^{13}C NMR (100 MHz, $CDCl_3$) δ 159.9, 138.3 (dd, *J* = 33.3 Hz, 25.0 Hz), 125.8 (t, *J* = 9.3 Hz), 112.8 (t, *J* = 244.4 Hz), 76.5 (t, *J* = 32.8 Hz), 73.8, 70.5, 70.1, 33.2, 27.5, 22.5, 14.0; ^{19}F NMR (282 MHz, $CDCl_3$) δ −106.5 (dt, *J* = 292.2 Hz, 8.2 Hz, 1F), −108.5 (dd, *J* = 293.0 Hz, 19.5 Hz, 1F); IR (thin film) ν_{max} 2933, 2860, 1757, 1653, 1460, 1384, 1255, 834, 777 cm^{-1}; MS (ESI) *m/z* 298 $(M+NH_4)^+$, 335 $(M+Na+CH_3OH)^+$; HRMS Calcd for $C_{12}H_{18}F_2O_5Na$: 303.1015; found: 303.1021.

(*R*)-5,5-Difluoro-6-((1*S*,2*R*,3*S*)-1,2,3-trihydroxyheptyl)-5,6-dihydropyran-2-one (7)

Compound **7** was prepared from compound **15b** (100 mg, 0.23 mmol) using the same conditions as described for compound **15a**; yield: 60 mg (93%); clear oil.

$[\alpha]_D^{27}$ = −10.6 (*c* 0.50, $CHCl_3$); ^{1}H NMR (300 MHz, $CDCl_3$) δ 6.77 (m, 1H), 6.33 (d, *J* = 9.9 Hz, 1H), 4.81 (ddd, *J* = 14.4 Hz, 8.4 Hz, 5.1 Hz, 1H), 4.20 (m, 1H), 3.80 (m, 1H), 3.70 (m, 1H), 2.91 (brs, 3H), 1.57 (m, 2H), 1.35 (m, 4H), 0.92 (t, *J* = 6.6 Hz, 3H); ^{13}C NMR (100 MHz, $CDCl_3$): δ 160.2, 137.2 (dd, *J* = 40.2 Hz, 36.2 Hz), 126.5 (t, *J* = 10.8 Hz), 112.2 (dd, *J* = 324.3 Hz, 317.0 Hz), 79.5 (dd, *J* = 42.8 Hz, 37.0 Hz), 72.7, 72.0, 70.5, 33.3, 27.7, 22.5, 14.0; ^{19}F NMR (282 MHz, $CDCl_3$) δ −98.6 (dd, *J* = 289.6 Hz, 8.3 Hz, 1F), −109.5 (ddd, *J* = 290.7 Hz, 14.7 Hz, 4.2 Hz, 1F); IR (thin film) ν_{max} 2933, 2860, 1757, 1653, 1460, 1384, 1255, 834, 777 cm^{-1}; MS (ESI) *m/z* 298 $(M+NH_4)^+$, 335 $(M+Na+CH_3OH)^+$; HRMS Calcd for $C_{12}H_{18}F_2O_5Na$: 303.1015; found: 303.1017.

gem-Difluoromethylenated boronolide (4)

To a solution of **5** (30 mg, 0.11 mmol) in CH_2Cl_2 (3 mL), Et_3N (0.2 mL, 1.6 mmol), DMAP (3.9 mg, 0.033 mmol) followed by Ac_2O (55 mg, 0.55 mmol) was added at 0 °C. The resulting mixture was warmed to room temperature and stirred overnight. The reaction was quenched with a saturated $NaHCO_3$ solution and extracted with CH_2Cl_2. The organic layer was washed with brine, dried over anhydrous Na_2SO_4, filtered and concentrated. The residue was purified by flash chromatography on silica gel (petroleum ether/ethyl acetate = 4:1) to afford compound **4** (37 mg, 85% yield) as a white solid, mp = 93–94 °C.

$[\alpha]_D^{22}$ = 7.4 (*c* 0.50, $CHCl_3$); ^{1}H NMR (300 MHz, $CDCl_3$) δ 6.77 (m, 1H), 6.31 (d, *J* = 10.5 Hz, 1H), 5.61 (dd. *J* = 7.8 Hz, 3.3 Hz, 1H), 5.45 (dd, *J* = 6.9 Hz, 3.3 Hz, 1H), 5.05 (m, 1H), 4.70 (ddd, *J* = 15.0 Hz, 9.6 Hz, 8.1 Hz, 1H), 2.14 (s, 3H), 2.12 (s, 3H), 2.07 (s, 3H), 1.28 (m, 6H), 0.89 (t, *J* = 7.2 Hz, 3H); ^{13}C NMR (100 MHz, $CDCl_3$) δ 170.4, 169.7, 169.0, 158.4, 137.7 (dd, *J* = 29.7 Hz, 27.4 Hz), 126.1 (t, *J* = 9.3 Hz), 112.2 (t, *J* = 242.2 Hz), 75.8 (t, *J* = 27.5 Hz), 71.5, 71.6, 66.5, 30.1, 27.0, 22.3, 20.8, 20.5, 13.8; ^{19}F NMR (282 MHz, $CDCl_3$) *δ* −106.6 to −106.7 (m, 2F); IR (thin film) ν_{max} 2923, 1785, 1650, 1374, 1067 cm^{-1}; MS (ESI) *m*/*z* 461 (M+ Na+CH_3OH)$^+$; HRMS Calcd for $C_{18}H_{24}F_2O_8Na$: 429.1332; found: 429.1347.

5-epi-_gem_-Difluoromethylenated boronolide (6)

Compound **6** was prepared from compound **7** (25 mg, 0.09 mmol) using the same conditions as described for compound **4**; yield: 30 mg (83%); clear oil.

$[\alpha]_D^{24}$ = −46.1 (*c* 0.85, $CHCl_3$); ^{1}H NMR (300 MHz, $CDCl_3$) δ 6.73 (m, 1H), 6.32 (d, *J* = 10.2 Hz, 1H), 5.59 (d, *J* = 6.6 Hz, 1H), 5.43 (dd, *J* = 6.9 Hz, 3.6 Hz, 1H), 5.11 (m, 1H), 4.91 (m, 1H), 2.11 (s, 6H), 2.06 (s, 3H), 1.27 (m, 6H), 0.87 (t, *J* = 7.2 Hz, 3H); ^{13}C NMR (100 MHz, $CDCl_3$) δ 170.6, 169.7, 168.9, 158.9, 136.6 (t, *J* = 28.3 Hz), 126.4 (t, *J* = 9.9 Hz), 111.8 (t, *J* = 238.2 Hz), 76.4 (dd, *J* = 32.7 Hz, 26.0 Hz), 71.6, 71.0, 66.1, 30.3, 27.0, 22.2, 20.6, 20.4, 13.7; ^{19}F NMR (282 MHz, $CDCl_3$) δ −100.1 (dt, *J* = 292.2 Hz, 9.0 Hz, 1F), −110.1 (ddd, *J* = 291.0 Hz, 14.1 Hz, 4.5 Hz, 1F); IR (thin film) ν_{max} 2961, 1753, 1559, 1375, 1221 cm^{-1}; MS (ESI) *m*/*z* 461 (M+Na+CH_3OH)$^+$; HRMS Calcd for $C_{19}H_{28}F_2O_9Na$: 461.1594; found: 461.1602.
